# A national propensity score-matched analysis of emergency laparoscopic versus open abdominal surgery

**DOI:** 10.1093/bjs/znab048

**Published:** 2021-03-16

**Authors:** P H Pucher, H Mackenzie, V Tucker, S J Mercer

**Affiliations:** 1 Department of General Surgery, Queen Alexandra Hospital, University Hospital Portsmouth NHS Trust, Portsmouth, UK; 2 Department of General Surgery, University Hospital Plymouth NHS Trust, Portsmouth, UK; 3 Department of Anaesthetics, Queen Alexandra Hospital, University Hospital Portsmouth NHS Trust, Portsmouth, UK

## Abstract

**Background:**

Laparoscopy has been widely adopted in elective abdominal surgery but is still sparsely used in emergency settings. The study investigated the effect of laparoscopic emergency surgery using a population database.

**Methods:**

Data for all patients from December 2013 to November 2018 were retrieved from the NELA national database of emergency laparotomy for England and Wales. Laparoscopically attempted cases were matched 2 : 1 with open cases for propensity score derived from a logistic regression model for surgical approach; included co-variates were age, gender, predicted mortality risk, and diagnostic, procedural and surgeon variables. Groups were compared for mortality. Secondary endpoints were blood loss and duration of hospital stay.

**Results:**

Of 116 920 patients considered, 17 040 underwent laparoscopic surgery. The most common procedures were colectomy, adhesiolysis, washout and perforated ulcer repair. Of these, 11 753 were matched exactly to 23 506 patients who had open surgery. Laparoscopically attempted surgery was associated with lower mortality (6.0 *versus* 9.1 per cent, *P* < 0.001), blood loss (less than 100 ml, 64.4 *versus* 52.0 per cent, *P* < 0.001), and duration of hospital stay (median 8 (i.q.r. 5–14) *versus* 10 (7–18) days, *P* < 0.001). Similar trends were seen when comparing only successful laparoscopic cases with open surgery, and also when comparing cases converted to open surgery with open surgery.

**Conclusion:**

In appropriately selected patients, laparoscopy is associated with superior outcomes compared with open emergency surgery.

## Teaser

Laparoscopy has positively impacted elective surgical outcomes but is sparsely used in emergency major surgery. This population-level analysis suggests laparoscopy may reduce mortality and length of stay compared to propensity score-matched laparotomy. Greater adoption of minimally invasive emergency surgery may improve patient care.

## Introduction 

Emergency laparotomy for bowel-related pathology is one of the most frequently performed and highest-risk operations. Over 50 000 emergency laparotomies are carried out each year in England alone, with a mean postoperative hospital stay of 16 days and a 30-day mortality rate of 9.6 per cent^1^. Patients undergoing emergency laparotomy continue to be at highest risk of death, and among the highest consumers of health system resources^2^.

While emergency laparotomy encompasses a range of operations (i.e. colectomy, adhesiolysis, irrigation and drainage), numerous risk-prediction frameworks^3–6^ have shown outcomes to be most strongly determined by physiology, associated pathology and degree of peritoneal soiling, rather than the type of procedure. The UK National Emergency Laparotomy Audit (NELA) was established to define this group more clearly and to improve care outcomes. However, despite this and other population-level quality-improvement initiatives^7^^,^^8^, outcomes for this patient group have only modestly improved in the past 5 years^1^.

Wide variations exist in the care of emergency laparotomy patients^1^. Laparoscopy has been identified as a potentially beneficial approach, with some data suggesting reductions in duration of hospital stay and postoperative mortality following major emergency bowel surgery^9^. Although well established in elective bowel surgery, the benefits of laparoscopy in emergency surgery are less known. Meta-analyses are largely limited to retrospective studies, with some suggesting potential benefits in limited applications, such as adhesiolysis^10^, but there are more conflicting results for other pathology, such as perforated gastroduodenal ulcers^11–13^.

Most recent reports show that uptake of emergency laparoscopy varies across the UK, ranging from 0–76 per cent^1^. On average fewer than 20 per cent of NELA-eligible cases are attempted laparoscopically, and half of these are converted to open surgery. This low rate of laparoscopy for emergency surgery may be due to system inadequacies and to individual surgeon preference^14^, but continues to be enabled by a lack of quality evidence demonstrating the potential benefits of the laparoscopic approach.

This analysis aims to assess the effect of laparoscopy on outcomes in emergency major general surgery at a population level.

## Methods

The NELA dataset is a prospective national database of all major (non-trauma related) emergency abdominal surgery in England and Wales. NELA’s inclusion criteria and recorded data have been previously described^1^; data analysis is permitted under the NHS Act 2006. This study is reported in accordance with the Strengthening the Reporting of Observational Studies in Epidemiology (STROBE) guidelines^15^.

Anonymized demographic, clinical and outcome data for all patients undergoing surgery between 1 December 2013 and 31 November 2018, were retrieved from the NELA database. Age data were grouped by 5-year intervals to preserve anonymity. P-POSSUM^5^, a well validated predictor of postoperative mortality risk, which combines diagnostic, demographic, biochemical and physiological variables, was used to stratify patients into low (0–4.9 per cent mortality risk), high (5.0–9.9 per cent), and very high (10 per cent or greater) risk, according to NELA-defined thresholds.

The NELA dataset defines the following surgical approaches: open, laparoscopic, laparoscopic converted to open, and laparoscopically assisted. As ‘laparoscopically assisted’ is not clearly defined by NELA, we elected to exclude these patients from analysis to ensure clear differentiation between laparoscopic and open procedures.

### Statistical analysis

Demographics between groups were compared using the Kruskal–Wallis test. Laparoscopically attempted cases (including converted) were matched exactly on a 2:1 basis with open cases using propensity matching with a tolerance of 0. Propensity scores were calculated with a logistic regression model; the dependent factor was surgical approach and the co-variables were all available potential predictors of surgical approach. These included age (categorized into 5-year intervals), gender, P-possum mortality (less than 5 per cent, 5–9.9 per cent, 10 per cent or greater), malignancy status (none, primary, nodal, metastases), peritoneal soiling (none, serous fluid, local pus, generalized contamination), surgical grade (consultant, non-consultant), and the most common operation types (perforated duodenal ulcer, small bowel resection, colectomy, adhesiolysis, washout, other).

An overall (intention to treat) comparison of laparoscopically attempted cases with their exact open matched cases was performed.

Two further subgroup analyses were performed. First, (per-protocol) an analysis of laparoscopic completed cases and their open matched cases, to examine the effect of laparoscopically completed surgery on outcome. Second, converted cases were compared to their open matched cases, to assess whether abandoned attempts at laparoscopy (conversion) might be associated with detrimental effects on outcomes, in case for example this group reflected a higher rate of iatrogenic injury with resulting conversion.

The primary outcome compared was in-hospital mortality, secondary outcomes were blood loss, intensive treatment unit stay and overall postoperative duration of hospital stay. Categorical variables were represented as percentages and compared using χ^2^ test. Continuous variables were represented as medians and inter-quartile range (i.q.r.) and compared using the Mann–Whitney U test or the Kruskal–Wallis test if there were more than two groups.

## Results

Some 118 355 patients undergoing major emergency abdominal surgery were recorded by NELA during the 5-year study interval. Patients who received ‘laparoscopically assisted’ surgery (1435 patients, 1.2 per cent of the total) were excluded.

Laparoscopy was attempted in 17 040 of 116 920 patients (14.6 per cent), and 7 915 of 17 040 (46.4 per cent) were converted to open. The remaining 99 880 (85.4 per cent) underwent open surgery. The rate of laparoscopic completed, converted and open cases for different operations is shown in *Table 1*.

**Table 1:** Frequency of most common surgical procedures and surgical approach.

Surgical procedure	Approach			Total
	Laparoscopic	Converted	Open	
**Colectomy**	2362 (5.6)	2732 (6.5)	36 755 (87.8)	41 849
**Adhesiolysis**	1911 (9.8)	1177 (6.0)	16 416 (84.2)	19 504
**Small bowel resection**	343 (1.8)	1472 (7.8)	17 091 (90.4)	18 906
**Perforated duodenal ulcer**	1000 (13.9)	486 (6.8)	5714 (79.4)	7200
**Washout**	1173 (20.2)	597 (10.3)	4037 (69.5)	5807
**Other**	2336 (9.9)	1451 (6.1)	19 867 (84.0)	23 654

Values in parentheses are percentages.

In unmatched comparisons, the laparoscopic group was younger, had lower risk of mortality, was less likely to have malignant pathology, had less contamination and was more likely to be operated on by a consultant surgeon (*Table 2*). In-hospital mortality rate, duration of hospital stay and intraoperative blood loss were also lowest the laparoscopic group.

**Table 2:** Demographics and outcomes of non-matched patients.

		**Open** ^*^	**Laparoscopic** ^*^	**Converted** ^*^	***P*** ^‡^
		(*n* = 99 880)	(*n* = 9125)	(*n* = 7915)	
**Gender**					
Male		51 506 (51.6)	4323 (47.4)	3882 (49.0)	0.074
Female		48 374 (48.4)	4802 (52.6)	4033 (51.0)	
**Age category (years)**					
18–25		2442 (2.4)	434 (4.8)	310 (3.9)	<0.001
25–30		1935 (1.9)	480 (5.3)	355 (4.5)	
30–35		2251 (2.3)	483 (5.3)	406 (5.1)	
35–40		2778 (2.8)	470 (5.2)	441 (5.6)	
40–45		3665 (3.7)	698 (7.6)	573 (7.2)	
45–50		5276 (5.3)	716 (7.8)	616 (7.8)	
50–55		6532 (6.5)	725 (7.9)	626 (7.9)	
55–60		7474 (7.5)	745 (8.2)	672 (8.5)	
60–65		8921 (8.9)	848 (9.3)	812 (10.3)	
65–70		11 921 (11.9)	897 (9.8)	816 (10.3)	
70–75		13 106 (13.1)	804 (8.8)	761 (9.6)	
75–80		13 084 (13.1)	716 (7.8)	634 (8.0)	
>80		11 313 (11.3)	629 (6.9)	496 (6.3)	
**p-POSSUM mortality risk**					
<5% (low)		38 951 (39.1)	5583 (61.4)	4335 (54.9)	<0.001
5–9.9% (high)		17 391 (17.5)	1533 (16.9)	1289 (16.3)	
≥10% (very high)		43 208 (43.4)	1979 (21.8)	2276 (28.8)	
Missing		330 (0.3)	30 (0.3)	15 (0.2)	
**Contamination**					
None		36 400 (36.6)	4370 (48.2)	2312 (29.3)	<0.001
Serous fluid		27 438 (27.6)	1932 (21.3)	1906 (24.2)	
Localized pus		9932 (10.0)	1089 (12.0)	1190 (15.1)	
Free contamination		25 708 (25.8)	1677 (18.5)	2480 (31.4)	
Missing		402 (0.4)	57 (0.6)	27 (0.3)	
**Malignancy**					
None		76 533 (76.9)	7282 (80.3)	6495 (82.3)	<0.001
Local		11 354 (11.4)	891 (9.8)	757 (9.6)	
Nodal metastases		4381 (4.4)	333 (3.7)	262 (3.3.)	
Distant metastases		7234 (7.3)	563 (6.2)	375 (4.8)	
Missing		378 (0.4)	56 (0.6)	26 (0.3)	
**Surgeon grade**					
Consultant		88 022 (88.1)	8307 (91.0)	7102 (89.7)	<0.001
Other		11 858 (11.9)	818 (9.0)	813 (10.3)	
**In-hospital mortality**					
Yes		12 480 (12.5)	372 (4.1)	554 (7.0)	<0.001
No		87 400 (87.5)	8753 (96.0)	7361 (93.0)	
**Blood loss (ml)**					
<100		49 007 (49.4)	6976 (77.1)	4204 (53.5)	<0.001
100–500		43 248 (43.6)	1873 (20.7)	3224 (41.0)	
501–1000		4460 (4.5)	130 (1.4)	288 (3.7)	
>1000		2395 (2.4)	70 (0.8)	149 (1.9)	
Missing		770 (0.8)	76 (0.8)	50 (0.6)	
**Duration of ITU stay (days)** ^†^		0 (0–3)	0(0–0)	0(0–2)	<0.001
**Duration of postoperative hospital stay (days)** ^†^		11 (7–20)	7 (4–12)	9 (6–16)	<0.001
**In-hospital mortality**					
Yes		12 480 (12.5)	372 (4.1)	554 (7.0)	<0.001
No		87 400 (87.5)	8753 (96.0)	7361 (93.0)	

* With percentages in parentheses unless indicated otherwise;

† values are median (i.q.r.). ITU, intensive treatment unit.

‡ Kruskal–Wallis test.

### Propensity matched analysis

Of the 17 040 laparoscopically attempted cases, 11 753 were matched exactly to 23 506 (1 : 2) open cases. Some 5876 of 11 753 (50.0 per cent) were completed laparoscopically and 5877 (50.0 per cent) were converted, and these were matched to 11 752 and 11 754 open cases, respectively. The matched demographic, diagnostic and mortality risk predictor variables are shown in *Table 3* (laparoscopic *versus* open) and *Table S1* (converted *versus* open and laparoscopic completed *versus* open).

**Table 3:** Demographics and outcomes of matched patients.

		Open*	Laparoscopic attempted*
		(*n* = 23 506)	(*n* = 11 753)
**Gender**			
Male		11** **342 (48.3)	5671 (48.3)
**Age category (years)**			
18–25		838	419
25–30		604	302
30–35		776	388
35–40		968	484
40–45		1108	554
45–50		1588	794
50–55		1878	939
55–60		1900	950
60–65		2106	1053
65–70		2620	1310
70–75		2726	1363
75–80		2418	1209
80–85		2180	1090
>85		1796	898
**p-POSSUM mortality risk**			
<5% (low)		13** **080 (55.6)	6540 (55.6)
5–9.9% (high)		3542 (15.1)	1771 (15.1)
≥10% (very high)		6884 (29.3)	3442 (29.3)
**Contamination**			
None		9692 (41.2)	4846 (41.2)
Serous fluid		5902 (25.1)	2951 (25.1)
Localized pus		2344 (10.0)	1172 (10.0)
Free contamination		5568 (23.7)	2784 (23.7)
**Malignancy**			
None		19** **676 (83.7)	9838 (83.7)
Local		2126 (9.0)	1063 (9.0)
Nodal metastases		610 (2.6)	305 (2.6)
Distant metastases		1094 (4.7)	547 (4.7)
**Surgeon grade**			
Consultant		22** **372 (95.2)	11** **186 (95.2)
Other		1134 (4.8)	567 (4.8)
**Operation type**			
Perforated ulcer		1916 (8.2)	958 (8.2)
Small bowel resection		3282 (14.0)	1641 (14.0)
Colectomy		7654 (32.6)	3827 (32.6)
Adhesiolysis		5690 (24.2)	2845 (24.2)
Washout		1436 (6.1)	718 (6.1)
Other		3528 (15.0)	1764 (15.0)

Values in parentheses are percentages.

Comparing all attempted laparoscopic cases (both successfully completed and converted to open) to open surgery, patients who received attempted laparoscopy demonstrated significantly improved outcomes, with reduced levels of blood loss (less than 100 ml, 64.4 *versus* 52.0 per cent, *P* < 0.001), duration of hospital stay (median 8 (i.q.r. 5–14) *versus* 10 (7–18) days, *P* < 0.001) and mortality rate (6.0 *versus* 9.1 per cent, *P* < 0.001) (*Table 4*).

**Table 4:** Comparison of outcomes for propensity score-matched attempted laparoscopic and open groups.

	**Attempted laparoscopic** ^*^	**Open** ^*^	*P*
	(*n* = 11 753)	(*n* = 23 506)	
**Intraoperative blood loss**			<0.001^‡^
<100 ml	7542 (64.1)	12** **124 (51.6)	
100–499 ml	3719 (31.6)	9836 (41.8)	
500–999 ml	308 (2.6)	938 (4.0)	
≥1000 ml	146 (1.4)	439 (1.9)	
Missing	38 (0.3)	169 (0.7)	
**ITU duration of stay (days)** ^†^	0 (0–2)	0 (0–2)	0.095^§^
**Postoperative duration of hospital stay (days)** ^†^	8 (5–14)	10 (7–18)	<0.001^§^
**In-hospital mortality**	709 (6.0)	2135 (9.1)	<0.001^‡^

* With percentages in parentheses unless indicated otherwise;

† values are median (i.q.r.). ITU, intensive treatment unit.

‡ χ^2^ test,

§ Mann–Whitney U test.

Patients whose surgery was successfully completed laparoscopically also demonstrated significantly improved outcomes when compared to patients who went straight to laparotomy, blood loss (less than 100 ml, 76.2 *versus* 57.1 per cent, *P* < 0.001), postoperative duration of hospital stay (median 7 (i.q.r. 4–12) *versus* 10 (7–17) days, *P* < 0.001), and in-hospital mortality rate (4.5 *versus* 8.2 per cent, *P* < 0.001) (*Table 5*).

**Table 5:** Comparison of outcomes for propensity score-matched laparoscopic and open groups.

	**Laparoscopic** ^*^	**Open** ^*^	*P*
	(*n* = 5876)	(*n* = 11 752)	
**Intraoperative blood loss**			<0.001^‡^
<100 ml	4463 (75.9)	6666 (56.7)	
100–499 ml	1270 (21.6)	4469 (38.0)	
500–999 ml	80 (1.4)	354 (3.0)	
≥1000 ml	44 (0.7)	181 (1.5)	
Missing	19 (0.3)	82 (0.7)	
**ITU duration of stay (days)** ^†^	0 (0–0)	0 (0–1)	<0.001^§^
**Postoperative duration of hospital stay (days)** ^†^	7 (4–12)	10 (7–17)	<0.001^§^
**In-hospital mortality**	265 (4.5)	963 (8.2)	<0.001^‡^

* With percentages in parentheses unless indicated otherwise;

† values are median (i.q.r.). ITU, intensive treatment unit.

‡ χ^2^ test,

§ Mann–Whitney U test.

Comparing patients who were converted to open surgery with those who went straight to laparotomy, the converted patients still performed better, with reduced blood loss (less than 100 ml, 52.6 *versus* 46.8 per cent, *P* < 0.001), duration of hospital stay (median 12 (i.q.r. 8–21) *versus* 14 (9–24) days, *P* < 0.001), and in-hospital mortality rate (7.6 *versus* 10.0 per cent, *P* < 0.001) (*Table 6*).

**Table 6:** Comparison of outcomes for propensity score-matched converted and open groups.

	**Converted** ^*^	**Open** ^*^	*P*
	(*n* = 5877)	(*n* = 11 754)	
**Intraoperative blood loss**			<0.001^‡^
<100 ml	3079 (52.4)	5458 (46.4)	
100–499 ml	2449 (41.6)	5367 (45.7)	
500–999 ml	228 (3.9)	584 (5.0)	
≥1000 ml	102 (1.7)	258 (2.2)	
Missing	19 (0.3)	87 (0.7)	
**ITU duration of stay (days)** ^†^	0 (0–2)	0 (0–2)	<0.001^§^
**Postoperative duration of hospital stay (days)** ^†^	12 (8–21)	14 (9–24)	<0.001^§^
**In-hospital mortality**	444 (7.6)	1172 (10.0)	<0.001^‡^

* With percentages in parentheses unless indicated otherwise;

† values are median (i.q.r.). ITU, intensive treatment unit.

‡ χ^2^ test,

§ Mann–Whitney U test.

**Fig. 1:**
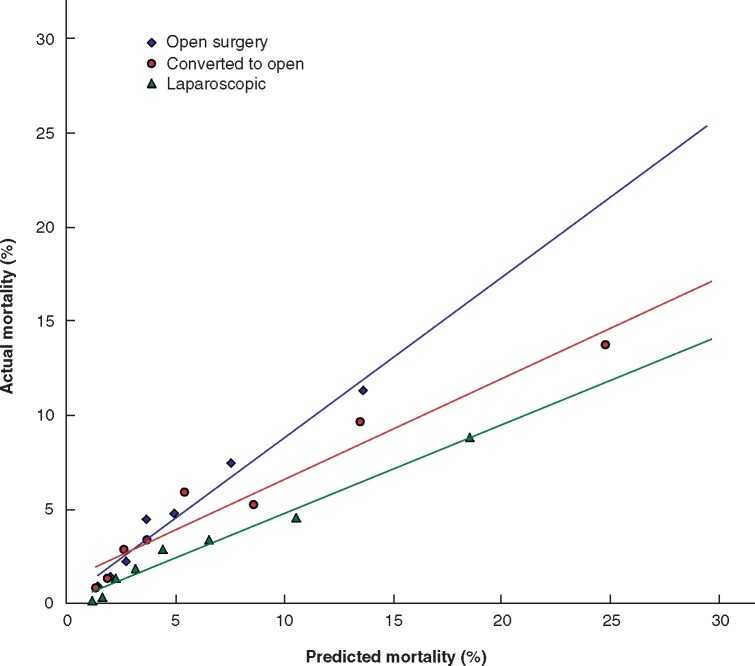
Calibration plot comparing observed *versus* P-POSSUM predicted mortality for patient deciles (1^st^ and 10^th^ outlier deciles excluded). Difference in mortality between groups *P* < 0.001 (Kruskal–Wallis test).

A calibration plot of observed vs. predicted mortality demonstrated divergent curves for the different approaches (*Fig. 1*).

## Discussion

This population-level analysis demonstrates the positive impact of laparoscopy in place of laparotomy on patient outcomes following emergency bowel surgery. When compared to a propensity score-matched cohort of patients, blood loss, duration of hospital stay and survival rates are improved. The reduction in mortality rate by almost half (8.2 per cent *versus* 4.5 per cent) and duration of hospital stay by 30 per cent (median 10 *versus* 7 days) far outweigh the benefits of any previous quality improvement measures in emergency surgery^7^^,^^8^^,^^16^, and suggest that a push to increase rates of laparoscopy could represent a potential step-change in quality of care.

It is important to recognize that these benefits are not applicable to all emergency cases. Selection bias in these cases is not only expected, it is necessary. Some pathologies lend themselves more to the laparoscopic approach than others: band adhesions in a virgin abdomen, or internal hernias following bariatric or colorectal surgery. Attempted laparoscopy for widespread faecal peritonitis from perforated diverticular disease or for resection of a large obstructing colonic tumour may, however, be impossible and pointless. Similarly, some patients are too physiologically unstable to permit anything other than a crash laparotomy and damage-control surgery.

What this study does illustrate is that the preferentially open approach practised in the majority of centres for patients that might have successfully undergone a minimally invasive procedure instead, potentially incurs negative implications for patient outcomes and hospital resource use. Laparoscopy in major emergency surgery is not only safe, but may be superior.

Furthermore, in this study, fears that laparoscopy might result in patient harm because of the perceived additional operative time or technical difficulty involved^17^, or through iatrogenic injury^18^, have not been substantiated. Considering all patients who underwent initial laparoscopy, these experienced reduced rates of blood loss, duration of hospital stay and in-hospital mortality compared with patients who underwent laparotomy, regardless of the eventual success or conversion of the operative approach. Starting laparoscopically, with its known differences to open surgery such as the necessary induction of pneumoperitoneum and greater use of potentially haemodynamically relevant Trendelenberg and reverse Trendelenberg positioning, did not impact these patients negatively. Similarly, patients converted from laparoscopic to open surgery still experienced improved outcomes compared with the laparotomy group, which may result from the benefit derived from a partial laparoscopic completion, and visual confirmation of the diagnosis allowing smaller and more targeted open incision. This study was unable to control for operative times, with potentially increased operative times sometimes cited as a negative factor of laparoscopy, but this did not affect the positive differences seen in outcomes for laparoscopy. Furthermore, eliminating the need to create and then close a large incision may in fact result in reduced operative time with laparoscopy, as was seen the recent LASSO randomized trial of laparoscopic versus open surgery for adhesional bowel obstruction^19^.

Existing literature on emergency laparoscopy remains relatively sparse, and of varying quality. The LASSO trial^19^, for example, demonstrated significantly reduced durations of stay after laparoscopy, but was not powered, with a total of 100 patients, to detect differences in morbidity or mortality. Others have reported improved outcomes across a number of indications such as colectomy^20^ or perforated ulcer repair^12^. This study represents the largest population-level analysis of emergency laparoscopy to date.

This study is limited by its retrospective nature and potential associated selection bias. Not all patients are suitable for laparoscopy, and not all variables relevant to this decision were captured. Surgeon experience and prior laparoscopic experience (for example, in elective practice), patient co-morbidity, prior surgery and body mass index may influence surgical decision-making but were not captured in this dataset, though multiple other factors were controlled for in this large cohort analysis through exact propensity score matching. The selection of P-POSSUM as a risk-adjusting metric was based on its well established validity and availability for the entire dataset. While the newer NELA mortality risk prediction algorithm^4^ has been since introduced, it is not yet widely validated, and was not available for the majority of patients in this dataset.

Long-term patient outcomes were not available, though given the reduced rates of incisional hernia or adhesions seen in other comparative studies of laparoscopy versus laparotomy^21^, the advantages reported in this study may eventually be even greater still through the avoidance of such longer-term sequelae of open surgery. Finally, this study aimed to assess the effect and potential benefit of laparoscopy but has not identified patient or pathological factors which determine the likelihood of surgical success via a minimally invasive approach. This remains the domain of the attending surgeon, with further research on this topic required.

Surgery remains a multidisciplinary endeavour. In particular, the importance of a quality radiology service cannot be overstated. Expert radiologists will not only diagnose intra-abdominal pathology more accurately^22^; they may be able to advise on the urgency of surgery, the degree of contamination, the presence of intra-abdominal adhesions and even the optimum laparoscopic port positions.

As individual surgeons and their emergency surgery teams perform more laparoscopic emergency surgery and become more confident in their skills, they extend their spectrum of what can be achieved laparoscopically. Skill and confidence can be developed as a team, if all surgeons can be encouraged to embrace the culture of laparoscopy for emergency surgery, and to educate each other with respect to operative skills and decision making. Previously published data show a steady increase in the proportion of emergency cases attempted laparoscopically over the first 5 years of the NELA data collection, from 37 towards 76 per cent^9^; UK national reports demonstrate a similar increasing trend, from 11 towards 20 per cent^1^.

Laparoscopy for emergency surgery must be considered safe, feasible in a much larger number of patients than currently practised, and potentially superior to open surgery. The significant improvements in mortality and length of stay mean that widespread implementation of the laparoscopic approach may be an important change to improve emergency surgery outcomes across the country, both for the individual patient, and for health systems as a whole.

## Supplementary Material

znab048_Supplementary_Data
