# Supplementary material for: A national propensity score-matched analysis of emergency laparoscopic versus open abdominal surgery
Source: Br J Surg. 2021 Mar 16;108(8):934–40. doi: 10.1093/bjs/znab048 (PMC10364898; doi:10.1093/bjs/znab048)
Supplement: znab048_Supplementary_Data [file znab048_supplementary_data.docx]

| **Supplementary table 1.** Demographics and outcomes of matched laparoscopic completed and converted patients (open matched cases have identical demographics). Reported as exact values (%) or median (IQR). | | | | | |
| --- | --- | --- | --- | --- | --- |
|  |  | **Laparoscopically completed** | | **Converted** | |
| n | | 5876 | | 5877 | |
| Gender | | | | | |
|  | Male | 2811 | | 2860 | |
|  |  | 47.8% | | 48.7% | |
|  | Female | 3065 | | 3017 | |
|  |  | 52.2% | | 51.3% | |
| Age category | | | | | |
|  | 18 - 25 |  | 200 |  | 219 |
|  |  |  | 3.4% |  | 3.7% |
|  | 25 - 30 |  | 158 |  | 144 |
|  |  |  | 2.7% |  | 2.5% |
|  | 30 - 35 |  | 191 |  | 197 |
|  |  |  | 3.3% |  | 3.4% |
|  | 35 - 40 |  | 240 |  | 244 |
|  |  |  | 4.1% |  | 4.2% |
|  | 40 - 45 |  | 273 |  | 281 |
|  |  |  | 4.6% |  | 4.8% |
|  | 45 - 50 |  | 403 |  | 391 |
|  |  |  | 6.9% |  | 6.7% |
|  | 50 - 55 |  | 470 |  | 469 |
|  |  |  | 8.0% |  | 8.0% |
|  | 55 - 60 |  | 479 |  | 471 |
|  |  |  | 8.2% |  | 8.0% |
|  | 60 - 65 |  | 527 |  | 526 |
|  |  |  | 9.0% |  | 9.0% |
|  | 65 - 70 |  | 646 |  | 664 |
|  |  |  | 11.0% |  | 11.3% |
|  | 70 - 75 |  | 677 |  | 686 |
|  |  |  | 11.5% |  | 11.7% |
|  | 75 - 80 |  | 579 |  | 630 |
|  |  |  | 9.9% |  | 10.7% |
|  | 80 - 85 |  | 550 |  | 540 |
|  |  |  | 9.4% |  | 9.2% |
|  | >85 |  | 483 |  | 415 |
|  |  |  | 8.2% |  | 7.1% |
| p-POSSUM mortality risk | | | | | |
|  | <5% (low risk) | 3431 | | 3109 | |
|  |  | 58.4% | | 52.9% | |
|  | 5 - <10% (high) | 893 | | 878 | |
|  |  | 15.2% | | 14.9% | |
|  | ≥10% (very high) | 1552 | | 1890 | |
|  |  | 26.4% | | 32.2% | |
| Contamination | | | | | |
|  | None | 2977 | | 1869 | |
|  |  | 50.7% | | 31.8% | |
|  | Serous fluid | 1413 | | 1538 | |
|  |  | 24.0% | | 26.2% | |
|  | Localised pus | 467 | | 705 | |
|  |  | 7.9% | | 12.0% | |
|  | Free contamination | 1019 | | 1765 | |
|  |  | 17.3% | | 30.0% | |
| Malignancy | | | | | |
|  | None | 4830 | | 5008 | |
|  |  | 82.2% | | 85.2% | |
|  | Local | 565 | | 498 | |
|  |  | 9.6% | | 8.5% | |
|  | Nodal metastases | 169 | | 136 | |
|  |  | 2.9% | | 2.3% | |
|  | Distant metastases | 312 | | 235 | |
|  |  | 5.3% | | 4.0% | |
| Surgeon grade | | | | | |
|  | Consultant | 5618 | | 5568 | |
|  |  | 95.6% | | 94.7% | |
|  | Other | 258 | | 309 | |
|  |  | 4.4% | | 5.3% | |
|  | Operation type |  | |  | |
|  | Perforated DU | 624 | | 334 | |
|  |  | 10.6% | | 5.7% | |
|  | Small bowel resection | 304 | | 1337 | |
|  |  | 5.2% | | 22.7% | |
|  | Colectomy | 1651 | | 2176 | |
|  |  | 28.1% | | 37.0% | |
|  | Adhesiolysis | 1779 | | 1066 | |
|  |  | 30.3% | | 18.1% | |
|  | Washout | 471 | | 247 | |
|  |  | 8.0% | | 4.2% | |
|  | Other | 1047 | | 717 | |
|  |  | 17.8% | | 12.2% | |
